# An Integrated Pump-Controlled Variable Coupler Fabricated by Ultrafast Laser Writing

**DOI:** 10.3390/mi14071370

**Published:** 2023-07-04

**Authors:** David Benedicto, Juan C. Martín, Antonio Dias-Ponte, Javier Solis, Juan A. Vallés

**Affiliations:** 1 Departamento de Física Aplicada, Instituto de Investigación en Ingeniería de Aragón (I3A), Facultad de Ciencias, Universidad de Zaragoza, Pedro Cerbuna 12, 50009 Zaragoza, Spain; dbenedicto@unizar.es (D.B.); jcmartin@unizar.es (J.C.M.); 2 Laser Processing Group, Institute of Optics (IO-CSIC), Serrano 121, 28006 Madrid, Spain; antonio.dias@alcyonphotonics.com (A.D.-P.); j.solis@io.cfmac.csic.es (J.S.)

**Keywords:** pumped nonlinear coupler, symmetric directional coupler, femtosecond laser writing, integrated optics, erbium and ytterbium co-doped waveguides

## Abstract

The design and fabrication of a integrated symmetric directional coupler dependent o the pumping power and operating at a 1534 nm wavelength is reported. The twin-core waveguide was inscribed into Er^3+^/Yb^3+^ co-doped phosphate glass by a femtosecond laser direct writing technique. By optical pumping, the coupling ratio can be modulated due to the changes induced in the refractive index of the material. The experimental results demonstrated that the coupling ratio can be tuned continuously from 100/0 to 50/50 by increasing the pump’s power from 0 to 350 mW. The developed twin-core coupler has promising applications for on-chip all-optical signal processing and communication systems.

## 1. Introduction

Integrated optics provide a powerful platform for developing complex miniaturized photonic devices [1,2,3]. Among the different techniques used for the fabrication of integrated waveguides, the femtosecond laser writing technique stands out due to its unique 3D fabrication capabilities [4,5]. This technique works by tightly focusing a femtosecond laser pulse inside the bulk of a transparent material, causing a permanent modification of the refractive index through nonlinear processes [6]. The fabrication of several integrated optical devices has been successfully demonstrated using this technique [7,8,9].

Optical couplers are one of the key building blocks for combining and splitting optical signals in integrated photonic circuits. Geometrically, a directional coupler consists of a pair of symmetrical waveguides placed in close proximity to each other, facilitating their interaction along a specific length. These devices operate by means of evanescent coupling between the two closely spaced waveguides, enabling the efficient transfer of energy between them. Given their significance as fundamental elements in integrated optical circuits, numerous examples of femtosecond laser-written directional couplers can be found in the literature [10,11,12,13], including asymmetric couplers [14], mode couplers [15] and polarization-insensitive directional couplers [16], among other recent devices. Generally, these devices are implemented in glasses where the cores are formed directly by an increase in the refractive index of the focal volume, known as Type I waveguides [17]. However, there is a growing trend of directional couplers being fabricated using Type II and Type III waveguides, especially in crystalline structures where achieving a positive increase in the index is challenging [18]. This enables the fabrication of directional couplers in active crystals [19,20,21,22].

Once the length has been fixed and the wavelength selected, conventional couplers offer a fixed coupling ratio. However, the functionalities of an integrated circuit may require dynamic alterations to accommodate different implementations. Thus, the capacity to modify the coupling ratio becomes particularly interesting.

There are different approaches used to effectively adjust the coupling ratio of a directional coupler. One simple method involves manipulating the incoupling conditions. In Ref. [23], a beam-splitter-type waveguide laser fabricated in a Yb^3+^-doped YAG by femtosecond laser writing was presented. By varying the incoupling position, they achieved a splitting ratio that could be adjusted from 5:95 to 85:15 through 50:50. However, the inclusion of a mechanically moveable component had a negative impact on the integration of the coupler into an optical circuit in conjunction with other fixed components.

Other approaches involve the exploitation of the substrate’s active properties. Such is the case in Ref. [24], in which they took advantage of the large LiNbO_3_ electro-optic coefficient to tune the coupling ratio. The authors reported the fabrication of depressed cladding waveguides using the femtosecond laser writing technique to create a 2 × 2 directional coupler within a lithium niobate crystal. The coupler was integrated with two deeply embedded microelectrodes on both sides of the interaction region, allowing the variation of the coupling ratio. By varying the applied voltage between 0 and 600 V, a broad range of coupling ratios was achieved. This approach, however, is not suitable for on-chip all-optical signal processing.

Finally, an approach not yet implemented in femtosecond laser-written integrated circuits is to take advantage of the nonlinear properties of the material to induce changes in its refractive index by applying optical pump powers. Nonlinear interactions can induce modifications in the power exchange, leading to nonlinear transmission characteristics that can be applied in optical processing applications. In the 1990s, in-depth investigations into the existence of a nonlinear phase shift in optically pumped erbium-doped fibers were carried out. When pumped at 980 nm, phase shifts of the light for wavelengths close to the vicinity of the resonant transition at 1530 nm (from the I13/2  4 to I15/2  4 states) were observed [25,26]. The study placed particular emphasis on twin-core erbium-doped fibers [27,28,29], in which the phase-shift could be indirectly measured through the variation in the power exchange between the cores. This research paved the way for the development of pumped nonlinear couplers (PNC) [30]. In these devices, the signal coupling ratio is controlled by the pumping laser’s power.

Extensive research has been conducted on the pump-induced nonlinear change in the refractive index in Er^3+^-doped and Yb^3+^-doped fibers since then [31], and a wide range of passive optical couplers [10,12,13] and active waveguide devices [32] have been successfully fabricated by ultrafast laser writing in bulk glass materials in recent years. However, to our knowledge, no reports exist on the fabrication of an Er^3+^/Yb^3+^-co-doped twin-core integrated coupler that is dependent on the pump’s power and uses femtosecond laser writing. Thus, this report represents the first instance of such a device being developed. The article’s organization is outlined below.

Section 2 provides an overview of the materials and fabrication methods used in the development of the device, as well as the characterization techniques utilized. In Section 3, the obtained coupling ratios are presented. Finally, Section 4 is dedicated to discussing and interpreting the results in detail.

## 2. Materials and Methods

Er^3+^/Yb^3+^ co-doped integrated waveguides are interesting for fabricating PNC due to the high resonant nonlinearity at the C band based on the transition of the energy level in Er^3+^ ions [33] and due to the enhancement of pump absorption achieved through the cooperative energy transfer mechanisms between Er^3+^ and Yb^3+^ ions [34]. When pumped at 980 nm, the pump absorption is achieved both between the I15/2  4 and I11/2  4 levels of the Er^3+^ ions and between the F7/2  2 and F5/2  2 levels of the Yb^3+^ ions. As the absorption cross-section of ytterbium is significantly higher than that of erbium, the pump absorption is enhanced due to co-doping with Yb^3+^. The excitation of the Er^3+^ ions is then achieved by energy migration. An Yb^3+^ ion from the F5/2  2 level undergoes de-excitation to the F7/22 level, while an Er^3+^ ion, initially at the I15/2  4 level, is excited to the I11/2  4 level. The migration of energy is mainly unidirectional due to the fast de-excitation of the Er^3+^ ions from the I11/2  4 level to the metastable energy level I13/2  4, in which the enhanced transition takes part. The signal propagation constant is modified both because of the gain and because of the resonant nonlinearity that enhances the modulation of the refractive index [30]. This type of nonlinearity is significantly larger than Kerr type nonlinearities [35]. This results in a change in the coupling between cores, and therefore in the coupling ratio of the coupler, which can be modified by varying the pump’s power.

The fabrication of an integrated coupler in an Er^3+^/Yb^3+^ co-doped glass by ultrafast laser direct writing is detailed below, as well as the experimental setup used for its characterization.

### 2.1. Fabrication of a Coupler by Femtosecond Laser Writing

A twin-core integrated coupler was inscribed in a phosphate glass by femtosecond laser writing. The glass primarily consisted of phosphorus pentoxide (P_2_O_5_) with lanthanum and potassium oxide (La_2_O_3_, K_2_O) glass modifiers, and minor amounts of Er_2_O_3_ and Yb_2_O_3_. Detailed information regarding the composition of the glass can be found in Table 1. A high lanthanum oxide content in phosphate glasses results in a significant variation in the refractive index (Δn), mainly due to the migration of La^3+^ ions and the out-diffusion of K^+^ ions [36,37]. Femtosecond laser irradiation also increases the local concentration of other lanthanides such as Er^3+^ and Yb^3+^ present in the composition of the glass [38]. By adjusting parameters such as the writing speed and pulse energy, it is possible to control changes in the material such as variations in the refractive index and modification of the diameter in the inscribed zone [39].

The fabrication of the device was carried out with a fs laser with a high repetition rate (Tangerine, Amplitude Systems) operating at a 1030 nm wavelength and a 500 kHz repetition rate, emitting pulses with a width of 350 fs and 520 nJ of energy. The beam was shaped with a slit (1.3 mm) before focusing 130 μm underneath the sample’s surface with an aspheric lens (NA of 0.68). The scanning speed was set to 0.06 mm/s. The waveguides were written from side to side of the sample with identical writing parameters, resulting in a twin-core device. Finally, the waveguide’s end facets were polished. Further details on the setup of the writing can be found in Refs. [36,40].

A complete characterization of a set of two-core waveguides with different core-to-core distances can be found in Refs. [41,42]. The determination of the core diameter and refractive index contrast was based on asymmetric excitation of the two-core waveguides and measurements of the distribution of the intensity at the output end. The active properties of the glass were obtained by signal enhancement measurements in a single-core waveguide. As important parameters, the two-core waveguide’s length was 9.35 mm, the cores’ diameters were 5.3 μm and the difference in the refractive index between the cores and the substrate is 6.3 × 10^−3^. The propagation losses of an individual waveguide, behaving as single mode for the third-window range when isolated, were 1.3 and 7.6 dB/cm for the power of the signal (1534 nm) and pump (980 nm), respectively. The manufacturer’s data for the concentration of Er^3+^ and Yb^3+^ ions in the bulk glass were 2.38 × 10^26^ and 4.95 × 10^26^ ions/m^3^, respectively, increasing up to 3.00 × 10^26^ and 6.25 × 10^26^ ions/m^3^ in the center of the guiding region. The proposed twin-core coupler was composed of a two-core waveguide with a core-to-core distance of 15 μm.

### 2.2. Experimental Setup

**Figure 1:**
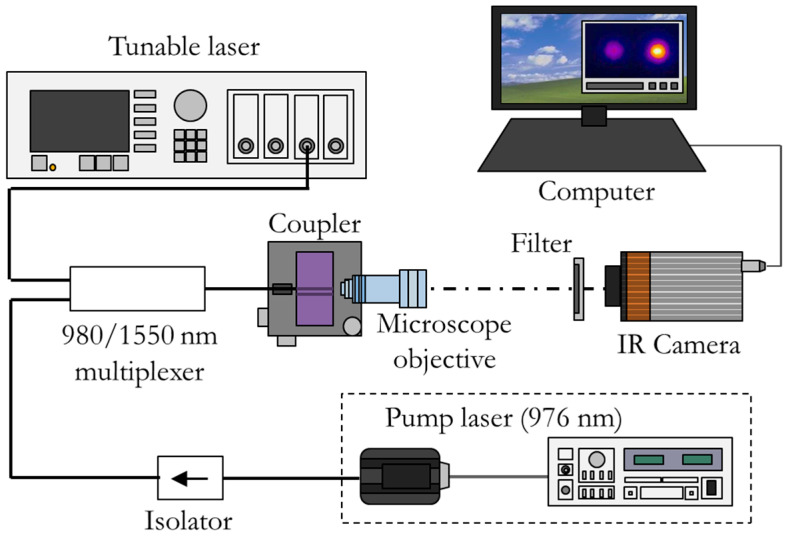
Experimental setup.

The experimental setup used for characterization of the device is depicted in Figure 1. The power of the input signal and the pump were multiplexed into a standard HI-1060 optical fiber. Its output end could be displaced to excite both cores of the integrated coupler. A microscope objective was placed at the coupler’s output end and the distribution of the intensity of the near-field output is registered with an infrared (IR) camera.

The signal’s power was provided by the third-window tunable laser module from the Agilent 8164B Lightwave Measurement System and the pump’s power was provided by the laser diode mount LDM-4980 controlled by an LDC-3900 module from ILX Lightwave. A filter (Thorlabs FEL1100) was placed between the objective (40 DIN, NA 0.65, achromatic) and the IR camera to block the output pump’s light (attenuation at 976 nm: 47 dB). The camera model used was the Bobcat-320 from Xenics.

This setup allowed the substitution of the microscope objective by a SMF (not shown in Figure 1) that led from the output power of the pump, signal and amplified spontaneous emission (ASE) to an optical spectrum analyzer (OSA) AQ6370Z from Yokogawa.

## 3. Results

### 3.1. The Coupling Ratio’s Dependence on Relative Incoupling Position

**Figure 2:**
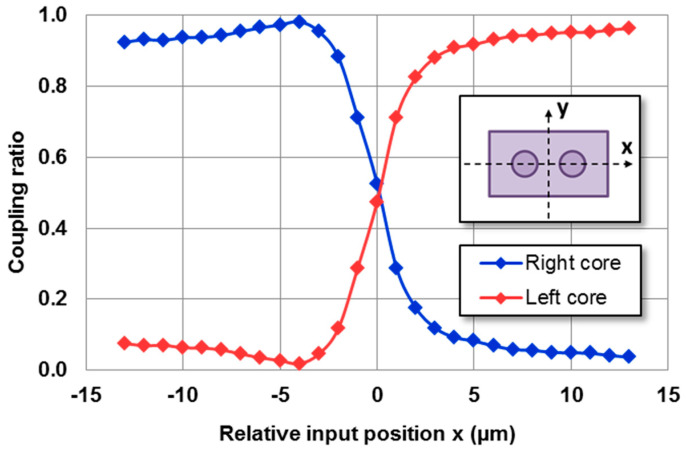
Measurements of the output coupling ratio as a function of the input fiber’s position. The signal’s input power was 0.1 mW for λ = 1534 nm. The legend specifies the output core used for calculating the coupling ratio.

In dual-core waveguides, the output coupling ratio depends on the distribution of the input intensity coupled to the waveguide. By modifying the incoupling conditions, the output coupling ratio can be varied. One simple approach to achieve this is by displacing the excitation fiber relative to the waveguide’s position. Figure 2 illustrates the coupling ratio of each core as a function of the transverse position of the excitation fiber, as shown in the schematic diagram.

The coupling ratio was defined as the output power of each core relative to the total output power. The measurements shown in Figure 2 were performed using the IR camera method. The image of the distribution of the output intensity was divided into two halves, each corresponding to one of the cores, and the coupling ratio was obtained through the intensity of each one of them.

The power ratio between both cores at the output of the directional coupler could be varied between 98/2 and 3/97 through 50/50 by simply displacing the input fiber by less than 20 μm in the transversal direction. This represents a modest improvement over the findings reported in [23] (5/95 to 85/15). However, the need for relative displacement between the components remains a significant obstacle in the implementation of a directional coupler within an integrated device. To overcome this challenge, a novel approach has been proposed.

### 3.2. The Coupling Ratio’s Dependence on the Optical Pump’s Power

**Figure 3:**
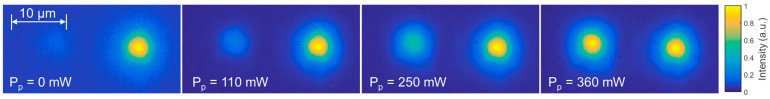
Experimental normalized distribution of the intensity of the output signal for the NPC under varying levels of the input pump’s power, P_p_ (labeled in each image). The feeding fiber directly excited the left-hand core with λ = 1534 nm and 0.1 mW of signal power.

A set of intensity profiles of the output signal under varying input pumping powers is displayed in Figure 3. The input signal’s power and the alignment of the feeding fiber with the left-hand core were kept constant across all measurements. A filter was placed before the IR camera so that the output pump’s power was blocked. The experimental results clearly demonstrated the impact of the pump’s power on the distribution of the intensity of the output signal, as expected in a PNC.

**Figure 4:**
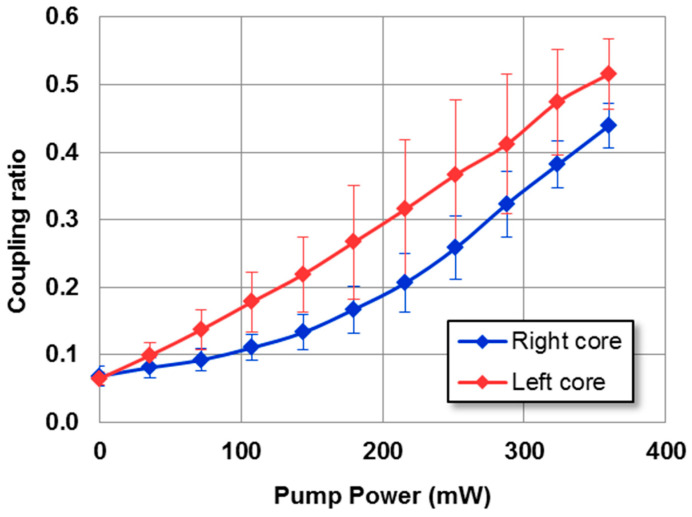
Experimental results for the coupling ratio of the NPC versus the pump power when the right (blue) or left (red) core was initially excited. The signal’s input power is 0.1 mW for λ = 1534 nm. The legend specifies the input core, which coincides with the output core used for calculating the coupling ratio.

To conduct a more comprehensive analysis and quantify the distribution of power, 10 series of measurements were performed, exciting in each one of the cores, and computing the mean average value of the output coupling ratio previously mentioned, together with its standard deviation. The results are shown in Figure 4.

As evident from the results, there was a slight variation in the coupling ratio when exciting the right or left core, even though the error bars overlapped at almost every point. This discrepancy can likely be attributed to a slight asymmetry between both cores due to inhomogeneities in the waveguides arising from the manufacturing process or impurities in the input facet of the waveguide that affected the light coupling within it.

The experimental uncertainty could be estimated by examining the error bars of the measurements, which ranged from 7% up to 32% of the mean value. This uncertainty primarily arose from variation in the initial coupling and the tolerances of the micrometer positioners. It is worth noting that even a slight variation of approximately half a micron, which aligned with the precision of the micrometer platforms (0.5 μm), could lead to variations in the coupling ratio with a similar magnitude to the error bars. However, it is important to highlight that the main sources of error were not inherent to the coupler itself but rather stemmed from its connection to the optical fiber. Therefore, integrating the directional coupler within an optical circuit has the potential to significantly diminish such errors.

**Figure 5:**
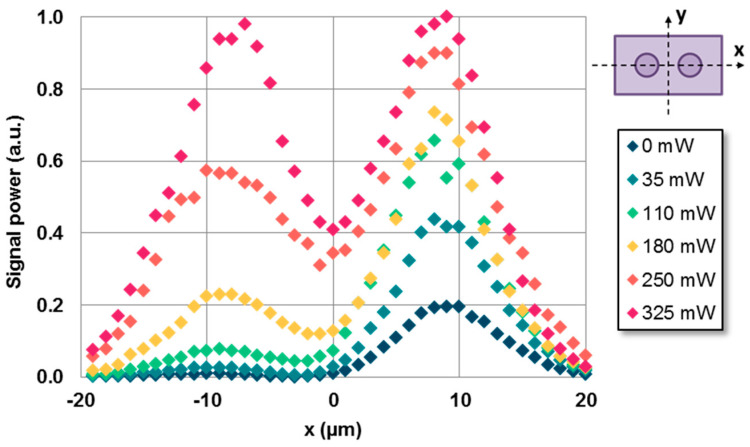
Experimental normalized signal power as a function of the output fiber’s position (*x*-axis, shown in the schematic illustration) for different values of pumping power. The left core was initially excited. The signal’s input power was 0.1 mW for λ = 1534 nm.

As the pump’s power increased, so did the signal’s power. In order not to saturate the IR camera, the time exposure needed to be re-adjusted every few measurements. Therefore, the current methodology used here presents limitations for gain measurements. Moreover, despite the pump’s power being blocked by a filter before reaching the IR camera, residual amplified spontaneous emission (ASE) may still be captured and not only the signal’s wavelength. To quantify the amplification of the signal’s power and avoid ASE, a second detection method was implemented, replacing the objective and camera with an HI-1060 fiber situated proximally to the waveguide’s output end. The output fiber could be displaced perpendicularly to both core axes, as illustrated in the schematic illustration shown in Figure 5 (*x*-axis). An OSA monitored the light coupled to the fiber, facilitating the selection of only the signal’s wavelength (λ = 1534 nm). The signal power’s dependence on the output fiber’s position (*x*-axis) for various values of pumping power is depicted in Figure 5.

**Figure 6:**
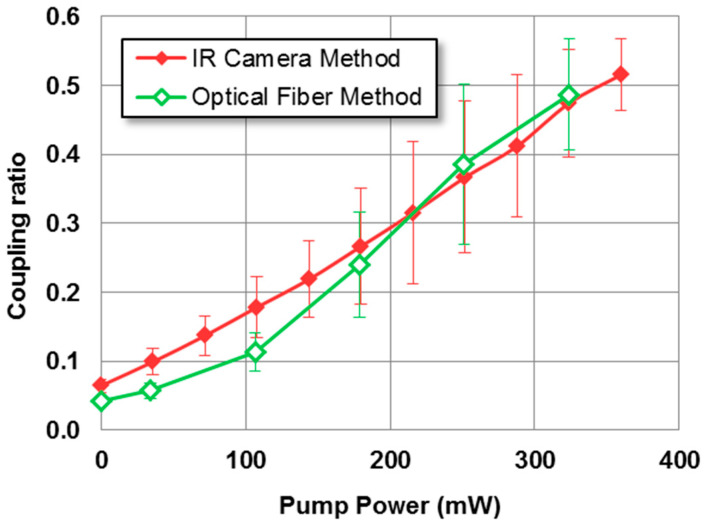
Comparison of the experimental coupling ratio of the NPC versus the pump’s power, depending on the method used (IR camera or optical fiber) when the left core was excited. The signal’s input power is 0.1 mW for λ = 1534 nm.

The coupling ratios obtained through the two methods, as compared in Figure 6, exhibited a high degree of similarity. The errors of the new measurements performed using the optical fiber method were estimated on the basis of the error percentages obtained from the IR camera method. This error may have been underestimated, as it did not account for the additional uncertainty in aligning the output fiber with respect to the directional coupler’s output end. However, considering the smoothness of the curves in Figure 5, it is expected that this error will be less significant compared with the one currently considered.

The results represented in Figure 6 showed a good overall agreement between the coupling ratios obtained using both methods, except for the values at a low pumping power. The good agreement between the results from both methods discarded any substantial influence of residual pumping power or fluorescence. The discrepancy between both methods for zero pumping power reflected the inherent differences between the two measurement techniques and the uncertainties in the measurement, which may have contributed to the observed discrepancies at low values of pumping power.

If we compare both techniques, the IR camera method, while faster and more direct, did not account for signal enhancement. On the other hand, the optical fiber method, which allowed a measurement of the enhancement, was more susceptible to experimental errors in alignment and required substantially more time.

With the imaging method with the IR camera, coupling ratios ranging from 0.05 up to 0.59 and 0.48, depending on whether the left or right core was excited, were obtained for pumping power values ranging from 0 to 360 mW. Similarly, the optical fiber method resulted in coupling ratios varying from 0.04 to 0.49 for pumping power values ranging from 0 to 325 mW. These results demonstrated the consistency and reliability of both methods for characterizing the pump-dependent coupler.

## 4. Discussion

As concluded in the previous section, both characterization methods yielded similar results for the coupling ratio of the NPC. If only modal coupling is of interest, which is typically the case of couplers, the IR camera method is sufficient. This method is faster and less susceptible to alignment errors. However, if the active behavior needs to be studied, the optical fiber method is necessary, as it allows for measurement of the signal’s enhancement. According to the results obtained by using both methods, it has been demonstrated that the NPC presented here enables continuous control of the coupling ratio. The power ratio between both cores at the output of the NPC can be varied between ~100/0 and 50/50 by adjusting the pump’s power from 0 to 350 mW. This is of significant interest for an optical directional coupler.

Exploring higher levels of pumping power beyond those used in this study may lead to more significant variations in the coupling ratio. However, on the basis of the measurements of the coupling ratio in other dual-core waveguides fabricated on the same substrate with different core-to-core distances [42], which exhibit a lower variation in the coupling ratio as a function of the input pump’s power, it is expected that the coupling ratio value will eventually stabilize as the pump’s power is increased further. Additionally, experiments were conducted by raising the signal’s power from 0.1 mW to 5 mW, yet no substantial changes were observed compared with the findings presented in the study. Those results consistently fell within the range defined by the error bars, indicating the limited impact of adjustments of the signal’s power on the measured coupling values. 

The fabrication of the NPC using an Er^3+^/Yb^3+^ co-doped glass matrix enables its integration within optical circuits that incorporate Er^3+^/Yb^3+^ co-doped amplifiers or highly efficient lasers, making it particularly useful for on-chip all-optical signal processing and communication systems. Additionally, the successful operation of the NPC has validated the effectiveness of the femtosecond laser writing technique for fabricating complex integrated photonic devices. It is worth noting that the effect of amplifying the signal’s observed in the coupler needs to be considered when used in practical applications, as is the case with other erbium-doped fiber nonlinear couplers (EDFNC).

This work presents a novel Er^3+^/Yb^3+^ co-doped twin-core integrated coupler dependent on the pump’s power and fabricated by femtosecond laser writing. To the best of our knowledge, this is the first report of a PNC of this type.

## Figures and Tables

**Table 1:** Composition of the 150M065A bulk glass manufactured by Glass Technology Services Ltd. (measured by X-ray Fluorescence (XRF) Spectroscopy).

Compound	Weight Percentage
P_2_O_5_	62.3
La_2_O_3_	17.3
Yb_2_O_3_	5.5
K_2_O	5.0
Al_2_O_3_	4.8
Er_2_O_3_	2.7
SiO_2_	2.4

## Data Availability

Data are available on request.
